# Validation of Current Good Manufacturing Practice Compliant Human Pluripotent Stem Cell‐Derived Hepatocytes for Cell‐Based Therapy

**DOI:** 10.1002/sctm.18-0084

**Published:** 2018-11-19

**Authors:** Samuel J.I. Blackford, Soon Seng Ng, Joe M. Segal, Aileen J.F. King, Amazon L. Austin, Deniz Kent, Jennifer Moore, Michael Sheldon, Dusko Ilic, Anil Dhawan, Ragai R. Mitry, S. Tamir Rashid

**Affiliations:** ^1^ Centre for Stem Cells and Regenerative Medicine King's College London London United Kingdom; ^2^ Institute for Liver Studies King's College Hospital, King's College London London United Kingdom; ^3^ Diabetes Research Group Faculty of Life Sciences & Medicine, King's College London London United Kingdom; ^4^ RUCDR Infinite Biologics Rutgers University New Brunswick New Jersey USA; ^5^ Stem Cell Laboratory, Department of Women and Children's Health Faculty of Life Sciences and Medicine, King's College London London United Kingdom

**Keywords:** Pluripotent stem cells, Hepatocytes, cGMP, Bioengineering, Liver therapy, iPSC, hESC, Hepatocyte differentiation, Cell transplantation, Cellular therapy

## Abstract

Recent advancements in the production of hepatocytes from human pluripotent stem cells (hPSC‐Heps) afford tremendous possibilities for treatment of patients with liver disease. Validated current good manufacturing practice (cGMP) lines are an essential prerequisite for such applications but have only recently been established. Whether such cGMP lines are capable of hepatic differentiation is not known. To address this knowledge gap, we examined the proficiency of three recently derived cGMP lines (two hiPSC and one hESC) to differentiate into hepatocytes and their suitability for therapy. hPSC‐Heps generated using a chemically defined four‐step hepatic differentiation protocol uniformly demonstrated highly reproducible phenotypes and functionality. Seeding into a 3D poly(ethylene glycol)‐diacrylate fabricated inverted colloid crystal scaffold converted these immature progenitors into more advanced hepatic tissue structures. Hepatic constructs could also be successfully encapsulated into the immune‐privileged material alginate and remained viable as well as functional upon transplantation into immune competent mice. This is the first report we are aware of demonstrating cGMP‐compliant hPSCs can generate cells with advanced hepatic function potentially suitable for future therapeutic applications. stem cells translational medicine *2019;8:124&14*

**Significance Statement**

Current good manufacturing practice (cGMP) compliant human pluripotent stem cells (hPSC) have been recently established for clinical application. To validate the capability of such cGMP lines for liver therapy, this article describes their proficiency on advanced hepatocyte production using both conventional culture plate and three‐dimensional hydrogel systems. The subsequent study on hepatocyte transplantation into immune competent mice using alginate encapsulation model further demonstrates the suitability of cGMP hPSC‐derived hepatocytes for cell‐based therapy. To the authors' knowledge, this is the first report demonstrating that cGMP‐compliant hPSCs can generate cells with advanced hepatic function potentially suitable for future therapeutic applications.

## Introduction

For patient's presenting with end‐stage chronic liver disease or acute liver failure (ALF), orthotopic transplantation of a donor liver remains the only curative treatment. Due to lengthening waiting lists and a severe scarcity of donors, mortality rates are increasing annually 1. As a result, an alternative strategy for treating these patients is urgently required. Allogenic transplantation of primary adult hepatocytes, the major functional cell‐type of the liver, is considered a viable solution in certain clinical indications 2, 3, 4. Lack of sufficient numbers of high‐quality hepatocytes; a result of isolating cells from tissue deemed unsuitable for transplantation, has limited the success of this programme 5, 6. Derivation of human embryonic stem cells (hESCs) 7 and their related induced pluripotent stem cells (hiPSCs) 8, 9, however, has generated growing optimism that the development of cellular therapies, such as would be suitable for liver disease, is finally an obtainable goal 10. Unlike primary hepatocytes, which cannot be cultured or expanded in vitro 11, 12, human pluripotent stem cells (hPSCs) possess an unlimited ability for self‐renewal 13. This capability to produce large batches of cells is of clinical significance as hepatocyte numbers approaching 8 billion may be required for transplant when correcting metabolic liver function in pediatric patients 14, or 15 billion to support liver failure in adults 15. Studies have shown that both hESCs 16, 17 and hiPSCs 18, 19, 20 can be differentiated into hepatocytes (hPSC‐Heps), sharing functional attributes of their in vivo equivalents, including albumin/α‐1 antitrypsin (A1AT) protein secretion, cytochrome P450 activity and glycogen storage. As research tools, hPSCs have delivered novel insights into human hepatic development 21, the creation of liver disease models 22, 23, and provided new platforms for pharmacological testing 24. Furthermore, the successful, albeit limited, repopulation of rodent livers following transplantation of both hESC, and hiPSC‐derived hepatocytes has been reported by several groups 25, 26, 27, suggesting hPSC‐Heps may be a viable treatment option for patients with liver disease.

The stem cell field has developed at an exceptional rate, which has resulted in the first human trials using cells derived from hESCs/hiPSCs being undertaken 28, 29, 30, 31, 32. Although approved for clinical use, these lines were in fact developed for research purposes and not produced under current good manufacturing practice (cGMP) guidelines. These manufacturing regulations for stem cell therapy products are described by the Food and Drug Administration in the U.S., and since the 2004 European Union Tissues and Cells Directive, the European Medicines Agency within the European Union. Generating cells under cGMP conditions ensures their clinical safety, and for cellular therapies should be differentiated in fully defined, xeno‐free conditions 33 to ensure reproducibility, and prevent xeno‐mediated infection or immune rejection 34.

In 2011, scientists from King's College London submitted the first xeno‐free clinical grade hESCs 35 to the U.K. Stem Cell Bank 36. More recently, cGMP‐compliant hiPSCs have been generated by teams in People's Republic of China 37, Japan 38, the U.S. 39 and the U.K. 40. These clinically compliant lines have been extensively characterized as pluripotent with profiles comparable to previously validated hPSCs derived outside of these manufacturing guidelines 41. However, given previous reports describing the varied potential for hPSC lines to differentiate into target cell types 42, 43, 44, evaluation of their hepatic differentiation potential represents an essential prerequisite to further clinical development work. The objective of this study was therefore to undertake this evaluation and then test the potential of differentiated cells to be engineered into three‐dimensional (3D) constructs suitable for clinical application.

## Materials and Methods

### Cell Lines and Cell Culture

Two cGMP hiPSC lines (CGT‐RCiB‐10 [line 1; Cell & Gene Therapy Catapult, London, U.K.] and LiPSC‐GR1.1 [line 2; Lonza, Walkersville, MD]) and one cGMP hESC line (KCL037 [line 3; Gifted from D. Ilic, King's College London]) were used in this study. All three lines were recovered into the culture conditions recommended by their respective suppliers. After two passages, each of the lines was subsequently maintained on Vitronectin XF (STEMCELL Technologies, Vancouver, BC, Canada) coated Corning Costar TC‐treated 6‐well plates (Sigma–Aldrich, St. Louis, MO) in TeSR‐E8 (STEMCELL Technologies, Vancouver, BC, Canada) and passaged every 4 days using Gentle Cell Dissociation Reagent (STEMCELL Technologies). Line 3 was passaged in TeSR‐8 supplemented with 10 μM Y‐27632 dihydrochloride (R&D Systems, Minneapolis, MN) to ensure cell survival.

**Figure 1:**
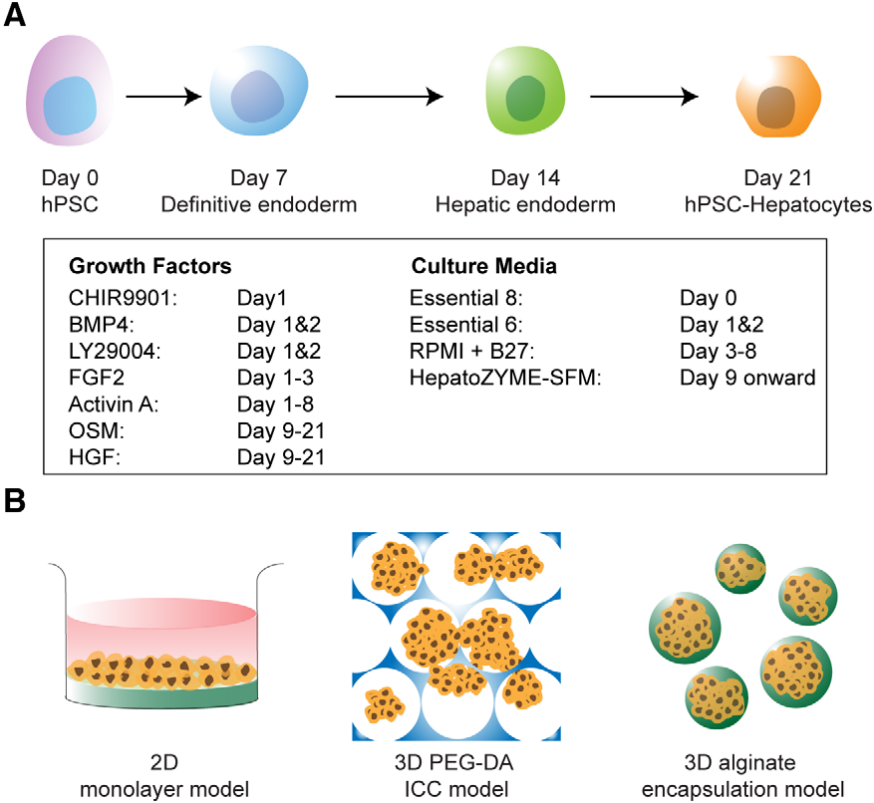
Generation of human pluripotent stem cell‐derived hepatocytes and their therapeutic potential in various model systems. **(A):** Four‐stage hepatic differentiation from human pluripotent stem cells (hPSC) to definitive endoderm, hepatic endoderm, and subsequently hepatocytes (hPSC‐Heps) over a 21‐day protocol by a defined cocktail of growth factors and small molecules (in the box). **(B):** Schematic of further maturation of hPSC‐Heps in two‐dimensional culture dish coated with type I collagen, three‐dimensional (3D) inverse colloidal crystal scaffold coated with type I collagen (inverse colloidal crystal) and encapsulation within 3D alginate microspheres.

Hepatocyte differentiation was carried out in Essential 6 Medium (Thermo Fisher Scientific, Waltham, MA; days 1–2), RPMI‐1640 Medium (Sigma–Aldrich; days 3–8) and HepatoZYME‐SFM (Thermo Fisher Scientific; day 9 onward) within Corning Falcon 100 × 20 mm style tissue culture dishes (Sigma–Aldrich). The following growth factors and small molecules were supplemented into the media for hepatocyte differentiation as shown in Figure 1A: 3 μM CHIR9901 (Sigma–Aldrich), 10 ng/ml BMP4 (R&D Systems), 10 μM LY29004 (Promega, Madison, WI), 80 ng/ml FGF2 (R&D Systems), 100 ng/ml and 50 ng/ml day 4 onward Activin A (Qkine, Cambridge, U.K.), 10 ng/ml OSM (R&D Systems) and 50 ng/ml HGF (PeproTech, Rocky Hill, NJ). Day 21 hPSC‐Heps were dissociated into a single‐cell suspension using TrypLE Express Enzyme (1×), no phenol red (Thermo Fisher Scientific).

### Brightfield and Immunofluorescence Imaging

Brightfield microscopy was performed on a Leica DMIL LED inverted microscope and imaged using the Leica DFC3000 G camera (Leica Microsystems, Wetzlar, Germany).

For immunofluorescence staining, samples were fixed for 10 minutes with 4% wt/vol paraformaldehyde, and then blocked and permeabilized in 1% wt/vol bovine serum albumin (Sigma–Aldrich), 3% donkey serum (Thermo Fisher Scientific) and 0.1% Triton X‐100 (Sigma–Aldrich). An additional 10 minutes of permeabilisation was performed using 0.5% Triton X‐100 for detection of nuclear antigens. Primary antibodies were applied for 1 hour and after wash steps Alexa Fluor‐555/488 conjugated secondary antibodies (Thermo Fisher Scientific) were incubated for 40 minutes. NucBlue Fixed Cell ReadyProdes Reagent (Thermo Fisher Scientific) was applied for visualization of cell nuclei. Imaging for two‐dimensional (2D) culture was performed on an Operetta High Content Screening System (PerkinElmer, Waltham, MA), and for 3D culture, a Nikon Eclipse Ti inverted spinning disk confocal microscope (Nikon, Minto, Japan).

### Real‐Time PCR

Total RNA was isolated using the RNeasy Mini Kit (QIAGEN, Hilden, Germany) according to manufacturer's protocol. RNA was quantified spectrophotometrically using the NanoDrop 2000 (Thermo Fisher Scientific). Three hundred and fifty nanograms of total RNA was used to produce first‐strand cDNA using the SuperScript VILO cDNA synthesis kit (Thermo Fisher Scientific). Quantitative real‐time PCR (RT‐PCR) was performed in a 10 μl reaction mixture consisting of cDNA, custom designed oligonucleotide primers (Sigma–Aldrich) and Fast SYBR Green PCR Master Mix (Thermo Fisher Scientific), on a CFX384 Touch Real‐Time PCR Detection System (Bio‐Rad, Hercules, CA). ACTB mRNA was used for housekeeping normalization.

### Flow Cytometric Analysis

Adherent cells were dissociated into a single‐cell suspension using TrypLE Express Enzyme (1×), no phenol red, and subsequently treated for 30 minutes with LIVE/DEAD Fixable Cell Stain (Thermo Fisher Scientific) and then fixed using 4% wt/vol paraformaladehyde (PFA) for 10 minutes. Cells were incubated with fluorophore‐conjugated antibodies for 30 minutes in the dark, and then washed twice with phosphate buffered solution (PBS). Immunophenotyping was carried out using the BD FACSCanto II system (Becton Dickinson, Franklin Lakes, NJ) and analyzed using FlowJo software (Becton Dickinson).

### Assessment of Hepatic Function

Albumin production of hPSC‐Heps was measured using the Human Albumin Quantification Set (Bethyl Laboratories, Montgomery, AL). Culture medium supernatants were collected after 48 hours and stored at −20°C. Enzyme‐linked immunosorbent assay (ELISA) was carried out according to manufacturer's instructions. Absorbance was measured at 450 nm on a Promega GloMax Multi+ Detection System plate reader (Promega).

Native cytochrome P450 *CYP3A4* activity was assessed using the *CYP3A4* P450‐Glo Assay with Luciferin‐IPA (Promega). The bioluminescent substrate was incubated on hPSC‐Heps for 1 hour before being collected for analysis. Luminescence was measured using a Promega GloMax Discover multimode microplate reader (Promega).

### Fabrication of ICC PEG‐DA Scaffolds

Thermo Scientific 4,000 Series monosized polystyrene beads of 100 ± 1.5 μm diameter (Thermo Fisher Scientific) were suspended in 70% EtOH and agitated using an ultrasonic bath. The dispersed bead suspension was seeded into hexagonal polypropylene molds and left to dry overnight on an orbital shaker.

A self‐standing colloidal crystal lattice was produced through annealing the beads at 120°C for 4 hours. Poly(ethylene glycol)‐diacrylate (PEG‐DA; Thermo Fisher Scientific) acrylate‐PEGN‐hydroxysuccinimide (Laysan Bio Inc., Arab) and Irgacure 2,959 photoinitiator (BASF, London, U.K.) were mixed together in dH_2_0 at a concentration of 50%, 10%, and 1% wt/vol, respectively. The bead lattices were placed within this precursor solution, and centrifugation (500*g*, 5 minutes) was performed to ensure complete infiltration. Hydrogel fabrication was completed through UV light induced gelation of the precursor solution and the polystyrene crystal lattice was removed from the scaffold through tetrahydrofuran (Sigma–Aldrich) soaking for 4 hours.

### Generation of hPSC‐Derived Hepatocyte Spheroids

A single cell suspension of day 21 hPSC‐Heps was prepared and 0.3 × 10^6^ cells were seeded per well of a 24‐well Aggrewell‐400 (STEMCELL Technologies). Aggrewell plates were prepared as recommended by the supplier. Centrifugation at 200*g* for 3 minutes was carried out to deposit cells into the microwells of the plate.

### Alginate Encapsulation of hPSC‐Derived Hepatocyte Spheroids

Encapsulation was performed as previously published 45, 46. In brief, spheroids were washed in saline before being resuspended into a final 1.8% ultra‐pure low‐viscosity, high‐glucuronic acid (≥60%), sodium alginate (FMC BioPolymer, Drammen, Norway) solution, which was then delivered by syringe pump through a 0.2 mm diameter nozzle, from which droplets were electrostatically deposited into a divalent cationic solution (1 mM BaCl_2_ + 50 mM CaCl_2_) to cause gelation.

### Live/Dead Staining

Fluorescine diacete (FDA; Sigma–Aldrich) and cell‐impermeant ethidium homodimer‐1 (EthD‐1; Thermo Fisher Scientific) were used as recommended by the supplier for staining of viable and dead cells. Spheroids and alginate encapsulated cells were incubated in 4 μM EthD‐1 for 35 minutes, washed with Hank's Balanced Salt Solution (HBSS) containing calcium (Thermo Fisher Scientific), then incubated in 50 μg/ml FDA for 90 seconds, and finally washed five times with HBSS before imaging on a Leica TCS SP8 Confocal laser scanning microscope (Leica Microsystems, Wetzlar, Germany).

### Transplantation of hPSC‐Derived Hepatocyte Spheroids

Alginate microencapsulated hepatocyte spheroids were intraperitoneally xenotransplanted into immune competent (C57BL/6 and Crl:CD1 [CD‐1]) and immune deficient (Rag2γ) mice. Spheroids were cultured in vitro for 3 days (CD‐1) or 7 days (C57BL/6 and Rag2γ) prior to encapsulation, and incubated within RPMI‐1640 medium for 2 hours before transplantation. Empty cell‐free microspheres were transplanted as a control. Surgical procedures were carried out under isoflurane anesthesia (1%–5% isoflurane, 95% oxygen, 1 l/min), with 30 μg/kg buprenorphine being administered immediately postsurgery. To create a sterile site of surgery, the mouse abdomen was shaved and cleaned with both antiseptic iodopovidone and isopropyl alcohol. A small incision through the skin, and a subsequent through the linea alba of the peritoneum allowed saline suspended alginate microspheres, containing approximately 2 × 10^3^ hepatocyte spheroids, to be delivered into the peritoneal cavity using a sterile pipette.

### Recovery of hPSC‐Derived Hepatocyte Spheroid Containing Microspheres

The mice were sacrificed by subcutaneous pentobarbital euthanasia 72 hours after transplantation. Blood samples were collected through cardiac puncture, and serum was diluted 1:10 for the detection of human albumin by ELISA. Injection of 5 ml saline into the peritoneal cavity was performed so that microspheres could be collected by peritoneal lavage. Microspheres were washed in saline and then maintained on ice, in RPMI‐1640 medium, until further analyses could be performed.

### Immunohistochemical Staining

Recovered microspheres were first fixed with 4% paraformaldehyde for 15 minutes, washed four times using PBS and transferred into 70% ethanol. The dehydrated samples were then paraffin infiltrated using Excelsior AS Tissue Processor (Thermo Fisher Scientific) and paraffin embedded using HistoStar Embedding Workstation (Thermo Fisher Scientific). Five micrometres thickness slides were then sectioned ready for immunohistochemical staining with a mouse and rabbit specific horseradish peroxidase/3‐amino‐9‐ethylcarbazole (HRP/AEC) detection immunohistochemistry (IHC) kit (Abcam, Cambridge, U.K.).

## Results

We firstly recovered two lines of hiPSCs, as well as one line of hESCs, each of which having been derived independently using cGMP‐compliant protocols. We maintained all lines in identical culture conditions comprising of xeno‐free cell culture matrix, Vitronectin, and chemically defined pluripotency culture medium, TeSR‐E8. After several passages within these culture conditions, each of the lines had fully reconditioned, with comparable cell morphologies and colony sizes (Supporting Information S1); each line producing characteristic rounded colonies, with small densely packed cells. We then began investigating the capability of each line in producing high quality hPSC‐Heps using an adapted four‐stage hepatocyte differentiation protocol based on Hannan et al. 47, and their potential application in downstream clinical applications by achieving advanced phenotypes in macroporous hydrogels and ensuring viability within transplantation ready cell encapsulation models for ALF therapy (Fig. 1).

### Differentiation of cGMP‐Compliant Stem Cells Toward hPSC‐Derived Hepatocytes

To prepare for hepatic differentiation, we seeded fragmented hPSC colonies obtained through enzyme‐free dissociation with minimal trituration onto gelatin coated tissue culture dishes. We treated hESCs with the small molecule Y‐27632 dihydrochloride, an inhibitor of the RHO/ROCK pathway, overnight to ensure sufficient cell attachment onto the gelatin. We found that the hiPSC lines did not require the addition of this small molecule to be passaged, or adhere to gelatin. We then initiated hepatic differentiation 2 days postseeding; having allowed enough time for the hPSCs to reestablish rounded colonies, with the outer cells tending to have a larger, more spread morphology.

**Figure 2:**
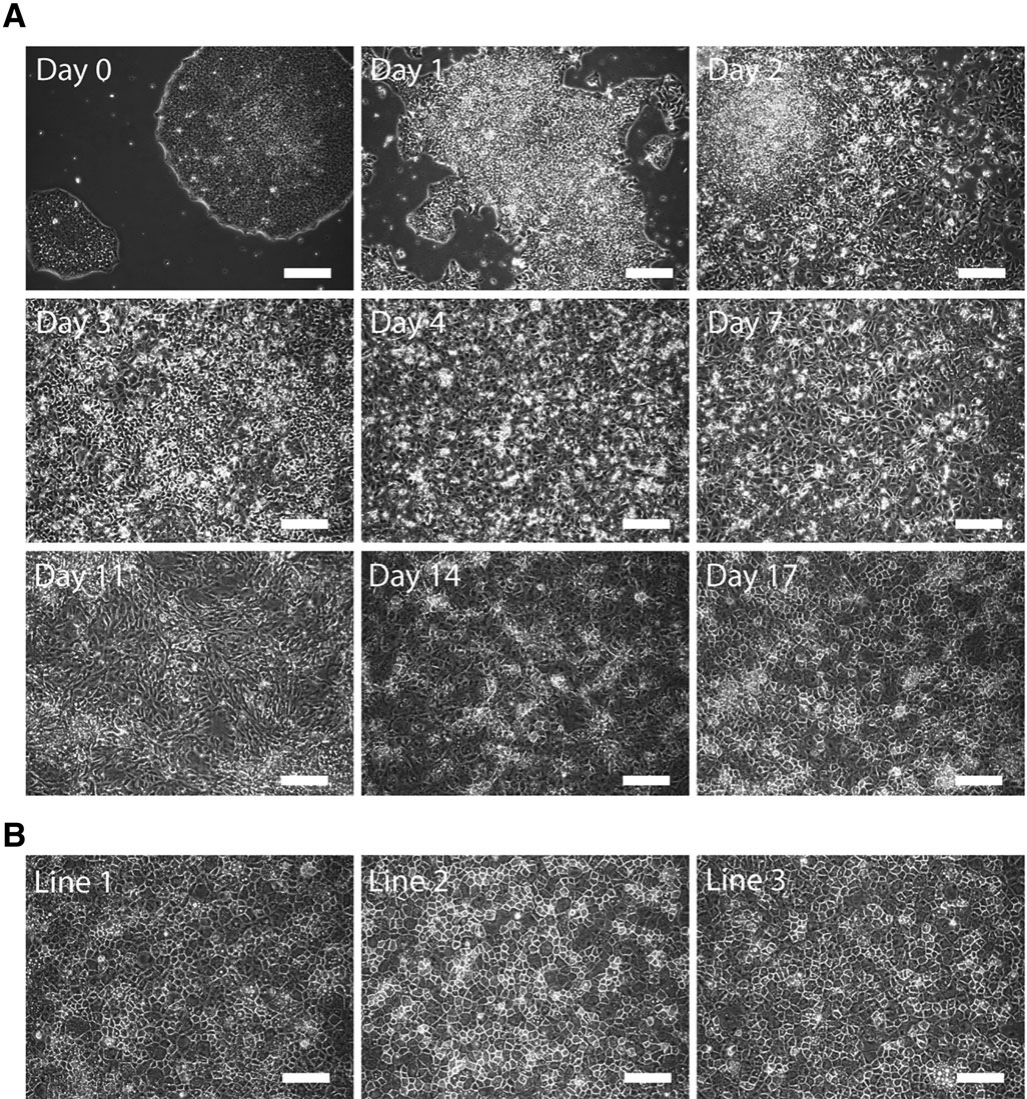
Morphological characterization of hepatic differentiated cells. **(A):** Brightfield microscopy images revealing the morphological transformation from day 0 pluripotent stem cell colony to day 17 polyhedral hepatocytes. **(B):** Representative images of day 21 human pluripotent stem cells‐Heps generated from three different current good manufacturing practice‐compliant lines. Scale bars: 100 μm.

We closely monitored the morphology of the cells throughout the differentiation and cross‐compared against that of our previous publication, that used non‐cGMP‐compliant hiPSCs, to ensure appropriate differentiation was achieved (Fig. 2A). Upon differentiation, the clear borders signifying stem cell colonies dissipate gradually with the peripheral cells starting to spread and migrate out sporadically by day 1 postdifferentiation. These protruding cells expand in size and proliferate to close the space between neighboring colonies until a confluent monolayer is formed by day 4. At this stage, cells exhibit definitive endoderm (DE)‐like morphology which persists until the media condition is changed to contain oncostatin‐M and hepatocyte growth factor at day 9. After this alteration, the morphology becomes more dynamic as the cells continue to differentiate. By day 14 the cells start to transform from their elongated morphology, observed at day 11, into a more cuboidal shape. The signature, well defined, polyhedral morphology of hepatocytes is observed across the whole culture by day 17. As demonstrated by the dynamic morphological transformation throughout the course of differentiation, all three of cGMP‐compliant lines appear capable of generating populations of DE, hepatic endoderm, and subsequently hPSC‐Heps (Fig. 2B).

### Characterization of cGMP‐Compliant hPSC‐Derived Hepatocytes

**Figure 3:**
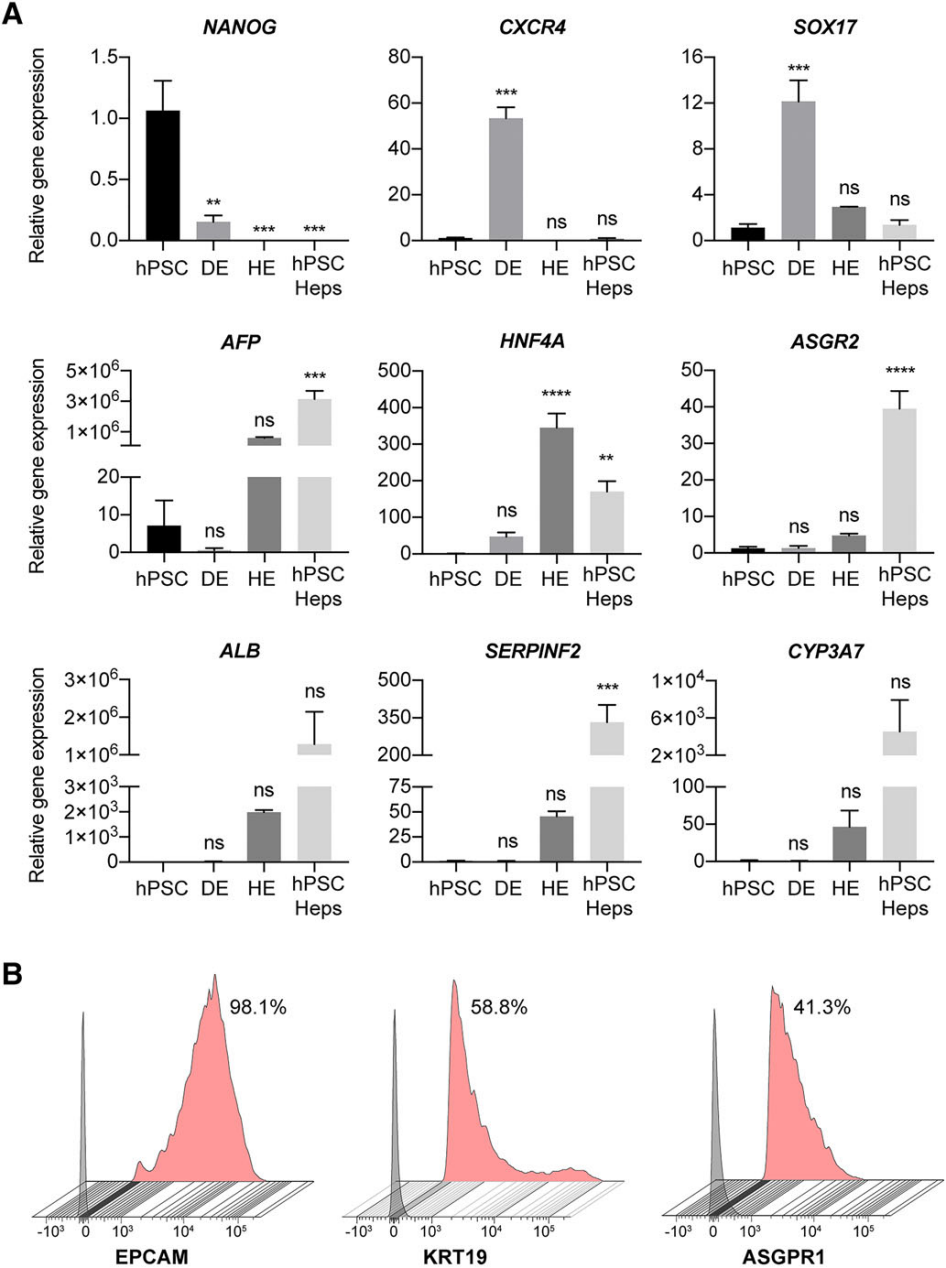
Phenotypical characterization of hepatic differentiated cells. **(A):** Differential expression of selected genes reveals progressive maturation of human pluripotent stem cells (hPSCs) to definitive endoderm, hepatic endoderm and then hepatocytes (hPSC‐Heps). Expression relative to the housekeeping gene, and normalized against the average expression of hPSCs; *n* = 3 experiments, 1 cell line. Data are mean ± SEM, ordinary one‐way ANOVA followed by Dunnett post hoc test to compare the mean of each group to hPSC expression. **, *p* < .005; ***, *p* < .0005; ****, *p* < .0001; ns: nonsignificant. Data shown for cell line 1. **(B):** Flow cytometry analysis of surface marker expression on hPSC‐Heps at day 21 of differentiation. Data shows the percentage of positive cells from the live cell population. Gray histogram represents fluorescence minus one (FMO) control used to establish the gate, red histogram represents stained hPSC‐Heps. All flow cytometry analysis is representative of at least three independent experiments. Data shown for cell line 3.

We next validated that the cGMP‐hPSCs were differentiating through the correct developmental lineage trajectory by collecting the cells and quantifying their mRNA expression at four distinct stages of the protocol (Fig. 3A). These time‐points represent undifferentiated hPSCs, DE (day 7), hepatic endoderm (HE, day 14) and finally hPSC‐Heps (day 21).

Importantly the expression of *NANOG*, a key pluripotency gene, is significantly downregulated upon differentiation, and is obsolete at day 21, the stage in which hPSC‐Heps have been produced. Furthermore, the expression of both *CXCR4* and *SOX17*, well established markers for both DE and primitive endoderm, are found to peak and be significantly upregulated at day 7 of differentiation when compared with undifferentiated hPSCs; after this time‐point the expression of both genes dissipates. To assess the correct differentiation into hPSC‐Heps, we selected six major hepatic genes for investigation. By day 21 a significant increase in the relative expression of *AFP*, can be measured. *AFP* is the gene encoding α‐fetoprotein, a major plasma protein produced by the developing liver and considered to be the fetal version of albumin 48. Incidentally, expression of *ALB*, albeit not significant, is detected at days 14 and 21 of differentiation, with the expression being greatest at the later time‐point. This provides evidence that at this stage the cell type produced is one that resembles a maturing hepatocyte. In addition, the relative gene expression for *HNF4A*, a hepatic transcription factor, as well as *ASGR2* (day 21 only), which encodes an asialoglycoprotein receptor isoform primarily found on liver cells, are significantly higher than that of undifferentiated or DE cells. In the case of *HNF4A*, the relative gene expression is peaked at day 14, indicative that the hepatocyte‐fate determination has occurred. Moreover, the expression for *SERPINF2*, the gene encoding the serpin α‐2 antiplasmin which is secreted in plasma by hepatocytes, is significantly elevated after 21 days of differentiation. Finally, nonsignificant, but elevated expression of *CYP3A7* is detected in day 21 hPSC‐Heps than the earlier time‐points. This cytochrome P450 3A family isoform is predominately expressed in the developing liver, with the translated enzyme involved in the metabolism of drugs, together with the synthesis of cholesterol, and various other lipids.

After validating the hepatic specific morphology and gene expression, we next sought to characterize the population profiles of the cells generated using our adapted protocol by performing flow cytometry analysis on day 21 hPSC‐Heps (Fig. 3B). The hepatic progenitor markers EpCAM (98.1%) and cytokeratin‐19 (58.8%) were expressed on most cells analyzed. In addition to these hepatic progenitor markers, expression of the asialoglycoprotein receptor 1 (ASGPR1), an endocytotic cell surface receptor specific to adult hepatocytes, was detected on 41.3% of day 21 hPSC‐Heps. The presence of hepatocyte progenitor markers and nonsignificant gene expression of *ALB* and *CYP3A7* displays the need for further maturation culture to produce a cell more closely resembling an adult hepatocyte.

### 2D Maturation of hPSC‐Heps

Having validated that cGMP‐compliant hPSCs were able to generate cells resembling immature hepatocytes when cultured in our differentiation conditions, we next aimed to advance their hepatic maturation through culturing the cells within different culture model systems to challenge the clinical relevance of our protocols. We first seeded the day 21 hPSC‐Heps onto collagen‐1 coated tissue culture plastic, as it is an extracellular protein that has been shown capable of supporting the long‐term culture, and liver‐specific functions, of isolated adult hepatocytes 49. Upon seeding, the hPSC‐Heps recovered their polyhedral morphology within 2 days. It should be noted that if not seeded to confluence, then many cells do not remain viable in 2D culture on collagen‐1, and those remaining do not proliferate, or go on to develop a mature phenotype.

**Figure 4:**
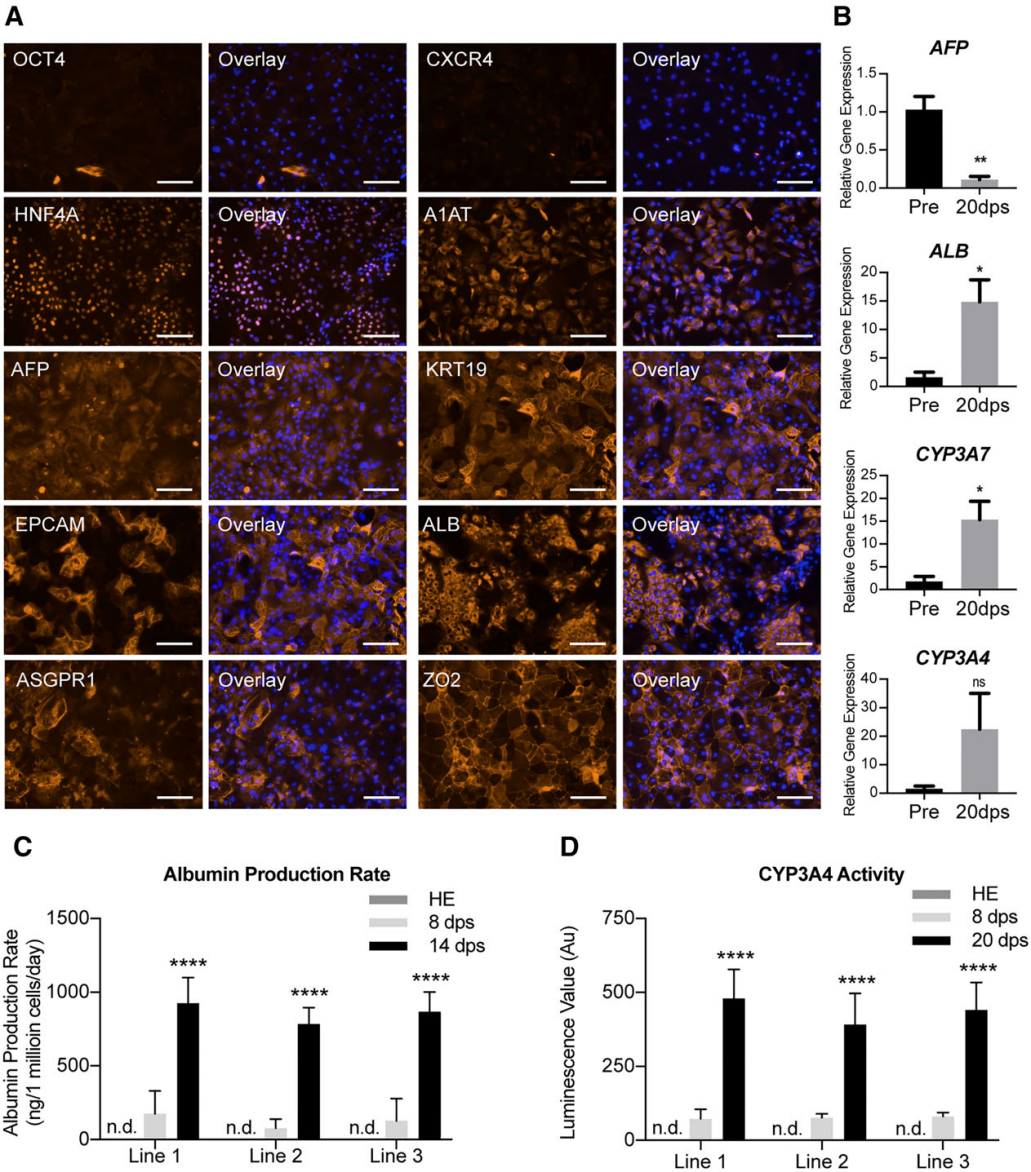
Characterization of human pluripotent stem cells (hPSC)‐derived hepatocyte maturation in two‐dimensional (2D) model. **(A):** Immunofluorescent images revealing the transition of pluripotency (OCT4), endodermal (CXCR4), and hepatic specification (*HNF4A*, A1AT, *AFP*, KRT19, and EPCAM) and mature hepatic (*ALB*, ASGPR1, and ZO‐2) expression in hPSC‐Heps after 20 days postseeding on 2D model. Representative images selected from each of the three lines. Scale bar: 100 μm. **(B):** Differential gene expression showing the relative expression of four key hepatic genes (*AFP*, *ALB*, *CYP3A7*, and *CYP3A4*) in preseeding (pre) and 20 days postseeding (20 dps) into 2D model. Statistical significance determined by Student's *t* test (two‐tailed); *n* = 3 experiments. Data shown for cell line 1. Data are mean ± SEM, *, *p* < .05; **, *p* < .005; ns: nonsignificant. **(C):** Albumin production rate of hPSC‐derived hepatic endoderm (HE), and hPSC‐Heps 8 and 14 days postseeding (8 and 14 dps) into 2D model; n.d.: not detected; *n* = 6 experiments per cell line. Data are mean ± SD, ****, *p* < .0001. **(D):** Cytochrome P450 3A4 enzyme activity of hPSC‐derived HE, and hPSC‐Heps 8 and 20 days postseeding into 2D maturation culture, n.d.: not detected; *n* = 3 (mean luminescence value [*n* = 6] of 3 independent experiments). Data are mean ± SD, ****, *p* < .0001.

After 3 weeks of maturation culture on collagen‐1 coated tissue culture plastic, we performed immunofluorescent staining to assess the hepatic maturity and heterogeneity of the hPSC‐Heps (Fig. 4A). Firstly, none of the pluripotency or endodermal markers, such as OCT4 and CXCR4 are detected in the cultures, which is a good indicator that the conversion of stem cell to hepatic lineage cell was completed in our protocol. It is encouraging to observe hepatocyte‐specific markers, for example the nuclear transcription factor, HNF4α and protease inhibitor, A1AT, to be abundantly expressed throughout the cultures. To further delineate the maturity of hPSC‐Heps cultured on collagen‐I coated 2D surfaces, we assessed the presence of hepatoblast specification (*AFP*, KRT19, and EpCAM) and hepatocyte specification (*ALB* and ASGPR1) markers, respectively. We found that even though the expression of mature markers, such as *ALB* and ASGPR1, are prevalent across the culture, we still observed substantial regions of cells that expressed *AFP*, KRT19, and EpCAM. The perseverance of these progenitor markers reveals a potential limitation of 2D culture in terms of differentiating a fully adult‐like hepatocyte. Of note, zona occluding 2 (ZO‐2), a component of tight junction proteins was highly expressed in 2D culture, highlighting the polyhedral morphology of the cells.

For a broader evaluation of the maturation of hPSC‐Heps in 2D, we compared the mRNA expression of cells 20 days postseeding to that of the preseeding population (Fig. 4B). A significant reduction in the level of *AFP* is achieved, while conversely, a significant enhancement in *ALB* expression occurs. A similar significant 15‐fold increase is found in the relative expression of *CYP3A7* after the additional 20 days of culture. Interestingly, although enzyme activity was measurable in the previous assay, the gene expression of *CYP3A4* is elevated, but not significantly higher than that of the preseeding population. These mRNA expression results corroborate with our immunostaining observation that our cells are yet to achieve advanced maturity in this 2D model system.

To characterize the liver specific functions of the cells, we monitored the albumin production rate (Fig. 4C and Supporting Information S2) and the metabolic activities of the cells over an extended period (Fig. 4D). As a 2D monolayer, hPSC‐Heps secreted albumin at levels detectable by ELISA after 6 days postseeding. Once cultured for a further week, the amount of albumin protein measured was significantly higher and an indicator of a maturing phenotype. Moreover, the albumin production is comparable between the three lines assessed, with no significant differences measured after 8 or 14 days of maturation. Furthermore, when cultured for an additional week (20 days postseeding), noninduced enzyme activity of cytochrome P450 3A4 can be detected (Fig. 4D). For each of the three lines, the enzyme activity detected is significantly greater at 20 days postseeding when compared with that of those after just 8 days. Again, no significant difference between the different cGMP‐compliant lines was measured at either time‐point, nor was activity detected in hPSC‐derived cells prior to 2D maturation culture. The presence of *CYP3A4* activity, which is initially absent from the liver of new‐borns, and responsible for >50% of medicinal drug metabolism, is a clear indicator that the extended culture period results in matured hepatocytes 50, 51.

### 3D Maturation of hPSC‐Heps Within a PEG‐DA‐Based Scaffold Suitable for Biomedical Applications

**Figure 5:**
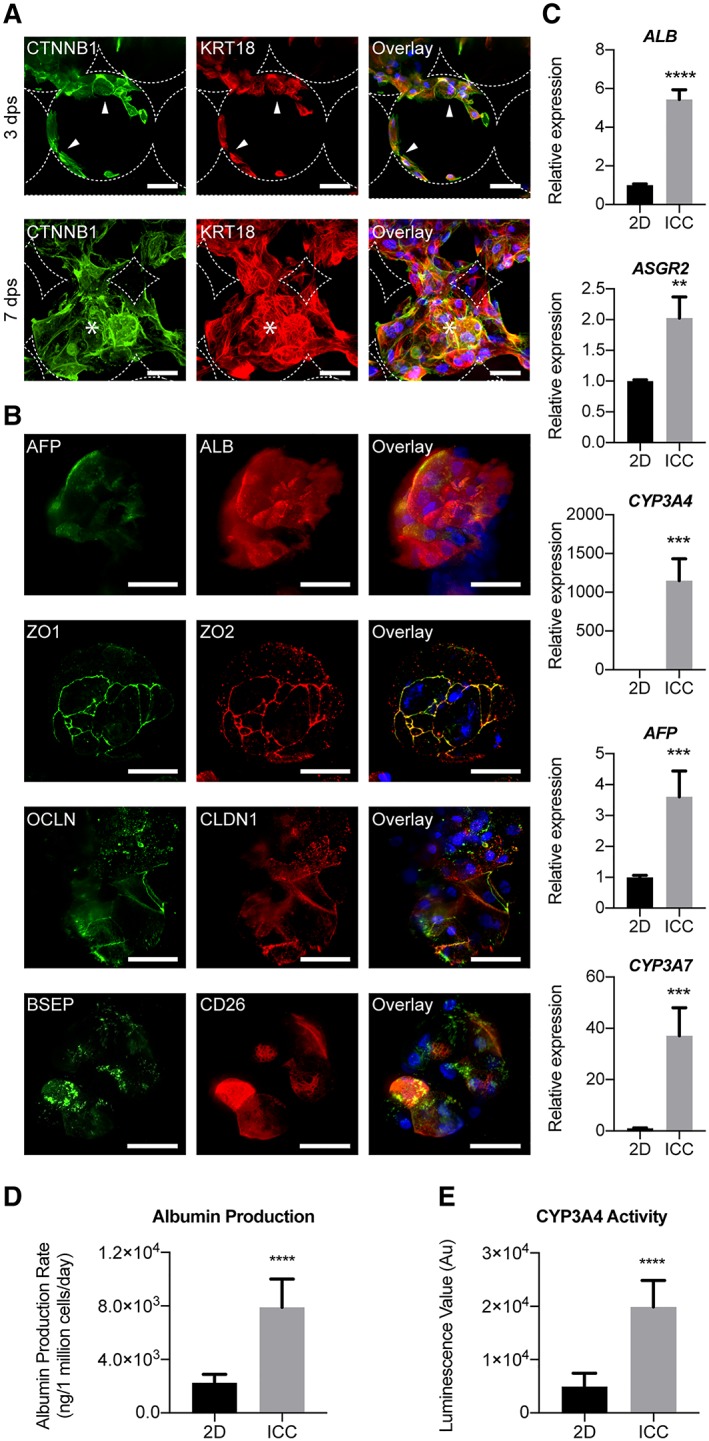
Characterization of human pluripotent stem cells (hPSC)‐derived hepatocyte maturation within three‐dimensional (3D) inverse colloidal crystal (ICC) model. **(A):** Immunofluorescent confocal images of hPSC‐Heps demonstrating two distinguished morphological phases inside the ICC scaffold. Three days postseeding an adhered lining across the hydrogel pores is observed, before the hPSC‐Heps morph into interconnected 3D clusters from 7 days postseeding onward. Arrowheads indicate cells lining the ICC scaffold surface; asterisks represent cells forming 3D clusters. Scale bar, 100 μm**. (B):** Immunofluorescent confocal images highlighting hepatic (*AFP* and *ALB*) and polarity (ZO‐1, ZO‐2, OCLN, CLDN1, BSEP, and CD26) proteins known to be present in adult human hepatocytes. Scale bar, 100 μm. Staining was performed on cell clusters after hPSC‐Heps had been cultured for 2 weeks in 3D. **(C):** Real‐time polymerase chain reaction showing the relative expression of five major hepatic genes (*ALB*, *ASGR2*, *CYP3A4*, *AFP*, and *CYP3A7*); *n* = 4 experiments, one cell line. **(D):** Albumin production rate of hPSC‐Heps cultured in 2D versus ICC models; *n* = 4 experiments, one cell line. **(E):** *CYP3A4* basal activity of hPSC‐Heps cultured in 2D versus ICC scaffolds; *n* = 4 experiments, one cell line. Data are mean ± SD. Student's *t* test (two‐tailed) analysis. **, *p* < .005; ***, *p* < .0005; ****, *p* < .0001. Data shown for cell line 1.

Having achieved considerable maturation on 2D collagen‐1 coated tissue culture plastic, we proceeded to load day 21 hPSC‐Heps into a more physiologically relevant model system for advanced hepatic maturation. We used a 3D macroporous PEG‐DA hydrogel, known as an inverse colloidal crystal (ICC) scaffold, that aims to mimic the anatomy of native liver tissue. The ICC scaffold has uniform sized pores, interconnected in a hexagonal pattern, and we have previously demonstrated the generation of liver organoids using both primary human fetal liver cells 52 and non‐cGMP‐compliant hPSCs 53 within this model. To facilitate the characterization of the morphogenic transformation of hPSC‐Heps within this scaffold, we performed a series of immunofluorescence staining and constructed the 3D images using confocal microscopy. Firstly, we observed that upon seeding into the ICC hydrogel, hPSC‐Heps establish cell‐matrix interactions with the coated ECM protein and achieve confluence over the concave surface of the internal pores of the scaffold within 3–5 days postseeding (Fig. 5A). Within 7 days postseeding, the cells self‐assemble into mechanically stable interconnected clusters that resemble organoid structures for up to at least 3 weeks in culture. These distinct phases of morphogenesis were further illustrated with immunofluorescence imaging of beta‐catenin (CTNNB1) and keratin 18 (KRT18) staining. Keratin 18 is the major intermediate filament protein in the liver, with a role in regulating glucose metabolism and modulating insulin signaling 54, whereas beta‐catenin is the central constituent of canonical Wnt signaling and is implemented as a fundamental regulator in hepatic physiology and development 55.

To further evaluate the phenotype of hPSC‐Heps within this ICC culture model, we selected additional hepatic specific markers, along with markers of hepatocyte polarity, for broader evaluation by immunofluorescence imaging. Closer inspection on confocal micrograph revealed that the self‐assembled interconnected clusters inside the ICC scaffold consisted of a heterogeneous population, with *AFP* positive liver progenitor cells occupying the periphery of the cluster and surrounding the *ALB* positive mature hepatocytes at the core (Fig. 5B). As most cells of the organoid‐like clusters stained positive for cytoplasmic albumin, amid only few positive for *AFP*, this staining served as confirmation that the seeded hPSC‐Heps go on to develop a more mature phenotype. Moreover, correctly localized expression of the tight junction proteins ZO‐1, ZO‐2, occludin and claudin‐1, bile‐salt efflux pump (BSEP) and dipeptidyl peptidase‐4 (CD26) were observed. These proteins are key components of bile canaliculi that form between the lateral faces of hepatocytes, and merge into bile ductules. The performed immunofluorescence staining provides validation of the liver‐specific signature of the organoid‐like structures generated.

We then performed a series of characterization to interrogate whether seeding hPSC‐Heps into this 3D environment resulted in the acquisition of greater maturity compared with the 2D culture model. RT‐PCR revealed that the expression of mature hepatic genes for proteins involved in biosynthesis (*ALB*), glycoprotein homeostasis (*ASGR2*) and metabolic functions (*CYP3A7* and *CYP3A4*) were significantly upregulated in the cells cultured within ICC scaffolds compared with those in 2D (Fig. 5C). Notably, the expression of fetal hepatocyte associated *AFP* was significantly higher in the 3D culture, however, this level is still significantly lower than in the day 21 cells initially seeded into the scaffold (Supporting Information S3).

Likewise, functional assays for albumin secretion (Fig. 5D) and *CYP3A4* enzyme activity (Fig. 5E) showed significant improvements for hPSC‐Heps matured within the ICC scaffold when compared with their 2D counterparts. The attainment and enhancement of these cellular functions are both major features of advanced hepatic differentiation, and their presence would be an unconditional necessity for any potential stem cell‐derived therapy. Cumulatively, these data confirm that cGMP‐compliant hPSC‐derived hepatocytes can successfully be matured into a functional hepatic phenotype within a 3D, readily up‐scalable, system. This combination of cGMP‐compliant cells and a biocompatible scaffold not only provides a platform conducive to the further study of hPSC‐Heps, but could also hold potential for future drug development and safety studies, and for assisting as a vehicle for cell transplantation.

### Generation of Alginate Encapsulated Hepatocyte Spheroids

To assess the broader translational potential of cGMP‐compliant hPSC‐derived hepatocytes for cell‐based therapies aimed at ALF, we carried out microencapsulation of hPSC‐Heps within alginate; a methodology that allows transplanted cells to be isolated from the recipient's immune responses 56. Successful encapsulation of hepatocytes within alginate hydrogels has been reported 57. However, to reduce the possibility of cell death during the encapsulation process 58, we used an additional established culture model, spheroid culture 59; known to both prolong viability 60 and phenotypes of hPSC‐Heps 61 (Supporting Information S4).

**Figure 6:**
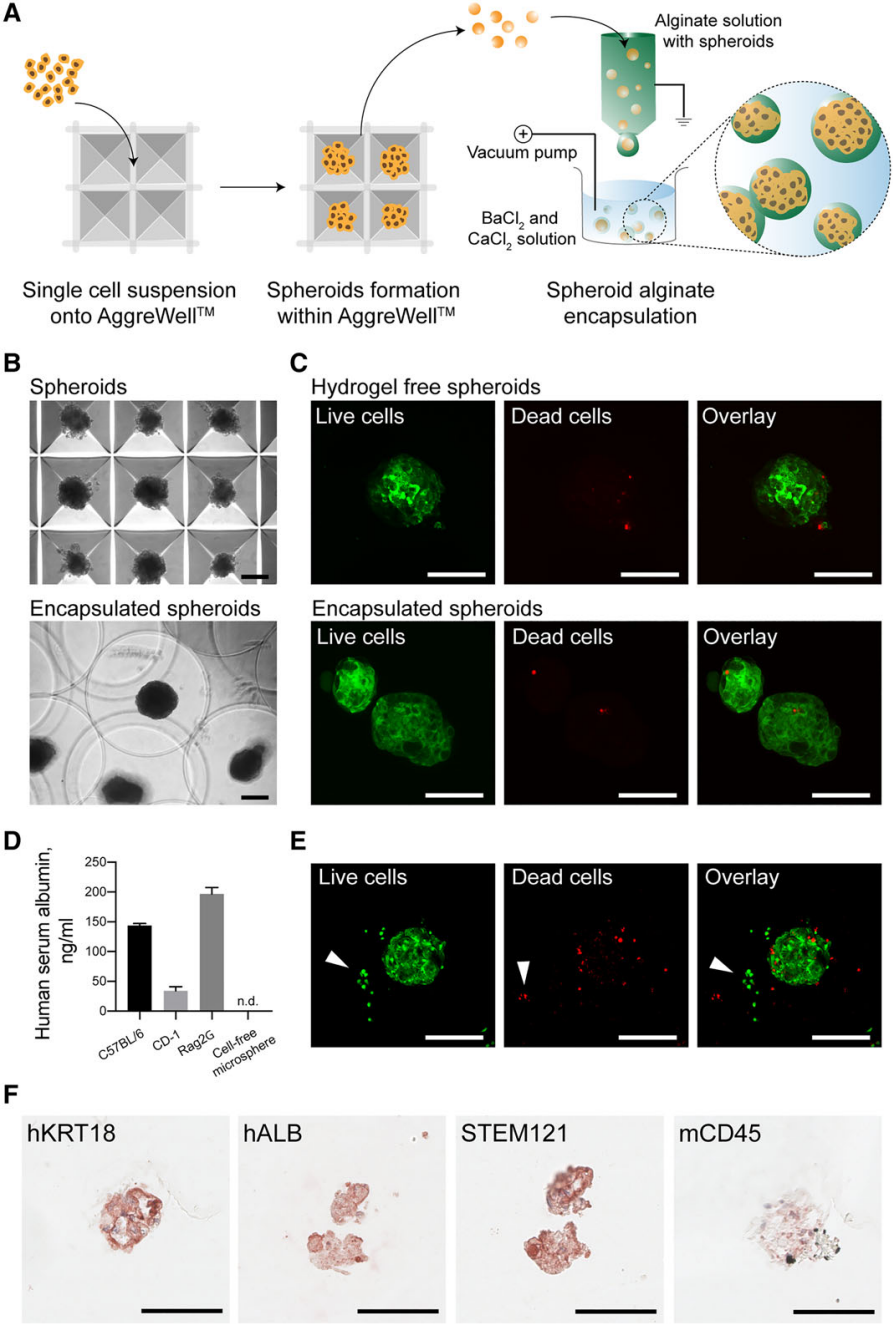
Generation of alginate encapsulated hepatocyte spheroids suitable for acute liver failure bridging therapy. **(A):** Schematic illustrating the high throughout generation of uniform hepatocyte spheroids made up from around 250 human pluripotent stem cells (hPSC)‐Heps using multi bioinert V‐bottom microwells and electrostatic alginate microsphere encapsulation within a BaCl_2_ and CaCl_2_ solution bath. **(B):** Brightfield images showing hepatocyte spheroids inside AggreWell microwells and alginate microsphere encapsulated spheroids. **(C):** Confocal images revealing the live/dead staining of hPSC‐Heps as hydrogel‐free spheroids, and within alginate microspheres, 6‐hours post encapsulation. **(D):** Human albumin detected within the blood serum of mice intraperitoneally xenotransplanted with alginate microspheres containing hepatocyte spheroids. **(E):** Confocal images revealing the live/dead staining of spheroids within microspheres recovered 3 days post‐transplantation. **(F):** Immunohistochemical staining of recovered microspheres showing cells positive for human hepatic markers (hKRT18 and hALB) human specific STEM121, and negative for murine/host immune marker (mCD45) at day 3 post‐transplantation. Data shown for cell line 2. Scale bars, 100 μm.

To generate spheroids for encapsulation (Fig. 6A), we used nonadherent microwell containing plates, called AggreWell, which have previously been used to generate spheroids from hiPSCs 62. We centrifuged single cell suspensions of hPSC‐Heps into microwells to facilitate cell–cell interactions for spheroid formation in large readily scalable quantities (Fig. 6B). The spheroids were left in culture for 7 days prior to encapsulation within 1.8% alginate microspheres. The 3D aggregates maintain their structure and uniformity as they are pumped through the microcapsule generator's 0.22 mm diameter nozzle at a rate of 10 ml/hour. The crosslinking of alginate occurs in under 5 minutes within barium chloride/calcium chloride solution, with most microspheres containing a single spheroid. We then washed the collected microspheres in saline and subsequently placed back into HepatoZYME‐SFM. To ensure that the viability of these hPSC‐Heps was preserved throughout this process, we carried out live/dead immunofluorescence staining using FDA and ethidium homodimer‐1 (EthD‐1) 6 hours after the encapsulation (Fig. 6C). Confocal microscopy confirmed that both alginate‐ and noncapsuled spheroids contain minimal to no dead cells (EthD‐1 stained nuclei), with close to all cells having hydrolysed FDA into fluorescent fluorescein. Furthermore, encapsulation does not impact the viability of hepatocyte spheroids placed back into further culture (Supporting Information S5).

To test the clinical suitability of alginate encapsulated hPSC‐Heps as a bridging therapy for ALF, we xenotransplanted spheroid containing microspheres into the peritoneal cavity of immune competent C57BL/6 and CD‐1 mice, as well as immune deficient Rag2γ mice. We monitored transplanted mice over a period of 12 days, and no signs of postoperative complication or mortality were observed. Blood sera collected from mice 3 days postprocedure revealed that the cells remain functional upon transplantation as the presence of human albumin (Fig. 6D) was detected by ELISA in all animals having received microspheres containing cGMP‐hPSC‐derived hepatocyte spheroids. Additionally, we recovered transplanted microspheres at this time‐point to evaluate in vivo cell survival and hepatic phenotype preservation. Microspheres were found dispersed throughout the peritoneal cavity with no sign of bruising, inflammation, or fibrosis (Supporting Information S6A). Upon closer inspection, we observed that microspheres recovered from each mouse strain had remained mechanically intact in vivo*,* and detected no sign of host cell invasion into the alginate (Supporting Information S6B); demonstrating that the encapsulation had served as an effective barrier. Most importantly, the viability and hepatic phenotype of transplanted spheroids was preserved in vivo throughout the critical therapeutic window for an ALF bridging therapy. Live/dead immunofluorescence staining of recovered spheroids revealed a minimal number of nonviable cells (Fig. 6E), comparable to that of encapsulated cells maintained in vitro (Supporting Information S7). We performed immunohistochemical staining on recovered microspheres that confirmed that the encapsulated hepatocyte spheroids had remained positive for human KRT18 and *ALB.* Staining with STEM121 that reacts specifically to a human cytoplasmic protein, known to be expressed in various tissues including the liver, further confirmed that cells present within the microspheres were of human origin. Moreover, antibodies against murine CD45 revealed no host cell infiltration within the alginate (Fig. 6F and Supporting Information S8). Overall, this proof‐of‐concept study together with our recent publications on in vitro 63 and in vivo 64 alloimmune studies suggests that microspheres encapsulating cGMP‐compliant hPSC‐Heps are both safe and effective for cell‐based therapies aimed at ALF.

In summary, we report that a library of cGMP‐compliant hPSCs can be differentiated into hepatocytes by a chemically defined protocol, which is suitable for clinical implementation. We confirm that this development goes via an endoderm‐like stage, before immature hepatocytes are obtained after 3 weeks. When matured by further culture in either 2D, or 3D models, these hPSC‐Heps express proteins known to be present on adult hepatocytes, have improved hepatic gene expression, and go on to demonstrate key liver functions.

## Discussion

The manufacture of cGMP‐compliant hPSC lines and optimization of translationally relevant differentiation technologies, is essential for clinical application of hPSCs 65. We have, to our knowledge for the first time in this report, validated that a library of clinical grade cGMP‐hPSC can successfully be differentiated into hepatocytes in a chemically defined protocol. Cells generated in this way demonstrated genes, proteins, and hallmark functional characteristics of hepatocytes, but as shown by us and others previously 66, fell short of the benchmarks set by primary adult cells. By subsequently seeding these immature hepatocytes into bio‐engineered 3D scaffolds fabricated from FDA approved material, we were able to drive the cells into liver tissue functionally approximated to the standard needed for clinical efficacy.

Following maturation within our 3D scaffold, elevated expression of key hepatic genes, such as *ALB*, *CYP3A7*, and *CYP3A4* for example were all found to occur. *CYP3A7* was first deemed as being exclusively expressed in the developing fetal liver 67, but it is now known to be present in up to 88% of adult livers 68, 69. This elevation in expression is of importance because cytochrome P450 enzymes are essential for the metabolism of numerous endogenous compounds and drugs. The CYP3A sub family specifically makes up 30% of the adult liver's cytochrome P450 constituency 70 and metabolize half of marketed drugs 71. Importantly, the level of *CYP3A4* enzyme activity is also elevated in hPSC‐Heps within 3D ICC culture when compared with 2D; as is the albumin production rate of the cells. It is also important to highlight that the upregulation in hepatic functions, and the organoid‐like morphological transformation observed in cGMP‐compliant hPSCs, are well aligned with our previous 3D study using a more well‐established non‐cGMP‐compliant hiPSC line 53. The unique fabrication technique of our scaffold permits the scale‐up of this cell‐scaffold complex—up to containing the billions of cells required for human translation—and can be functionalized with different recombinant proteins, molecules or mechanical parameters. Cumulatively, these design features suggest such a scaffold could be the ideal carrier for delivering cGMP‐compliant hPSC‐Heps into patients.

Assuming delivery of suitable numbers of functionally optimized cGMP cells can be achieved as above, a further challenge for clinical application will be to deal with the potential allogenic immune rejection of the host 72. Alginate hydrogel microencapsulation provides hPSC‐derivatives with a physical barrier from the recipient's immune system, through enclosure within a naturally occurring anionic polymer. Alginate, typically obtained from brown algae, is considered ideal for biomedical applications due to its biocompatibility, low cost, and ease of gelation 73. Numerous cell types have successfully been encapsulated within alginate, including mesenchymal stromal cells 74, pancreatic islets 75, and human hepatocytes 76, while optimized GMP grade alginate encapsulation protocols have already been established for the transplantation of human hepatocytes to provide metabolic function in patients with ALF 63.

Status 1 ALF failure patients are assessed as having a life expectancy of hours, to a few days, without a liver transplantation 77. The median number of days from status 1 listing to death is just 5.5 with over a fifth of adults dying on the waiting list 78. The condition of patients with ALF can change significantly, even within 72 hours of being listed for transplantation 79 with 13.9% of patients requiring “superurgent liver transplantation” due to ALF having a spontaneous recovery 80. These clinical reports support the hypothesis that ALF is potentially reversible without the need for transplantation if the host liver can be given enough time to recover. Our study demonstrated cGMP‐derived hepatic constructs remained viable, and more importantly, functional within the peritoneal cavity of fully immune competent C57BL/6 and CD‐1 mice for a time period long enough to result in recovery from ALF if used in patients. This in turn strongly advocates for further development of hPSC‐hepatocytes as a clinical therapeutic 63, 81, 82, 83.

## Conclusion

We report here a library of clinical grade hPSCs manufactured under cGMP conditions amenable to reproducible hepatic differentiation, 3D culture, and alginate encapsulation that is potentially suitable for human application.

## Author Contributions

S.J.I.B., S.S.N.: concept and design, collection and/or assembly of data, data analysis and interpretation, manuscript writing; J.M.S., A.J.F.K., A.L.A., D.K.: collection and/or assembly of data; J.M., M.S., D.I.: provision of study material or patients; A.D.: concept and design, manuscript writing; R.R.M.: concept and design, collection and/or assembly of data, manuscript writing; S.T.R.: concept and design, financial support, manuscript writing, final approval of manuscript.

## Disclosure of Potential Conflicts of Interest

S.T.R. is a scientific founder, shareholder, and consultant for DefiniGen, Ltd. The other authors indicated no potential conflicts of interest.

## Supporting information

Appendix S1: Supplementary Data
