# Supplementary material for: Validation of Current Good Manufacturing Practice Compliant Human Pluripotent Stem Cell‐Derived Hepatocytes for Cell‐Based Therapy
Source: Stem Cells Transl Med. 2018 Nov 19;8(2):124–37. doi: 10.1002/sctm.18-0084 (PMC6344902; doi:10.1002/sctm.18-0084)
Supplement: Supplementary file 1 — Appendix S1: Supplementary Data [file SCT3-8-124-s001.pdf]

Supplementary Data

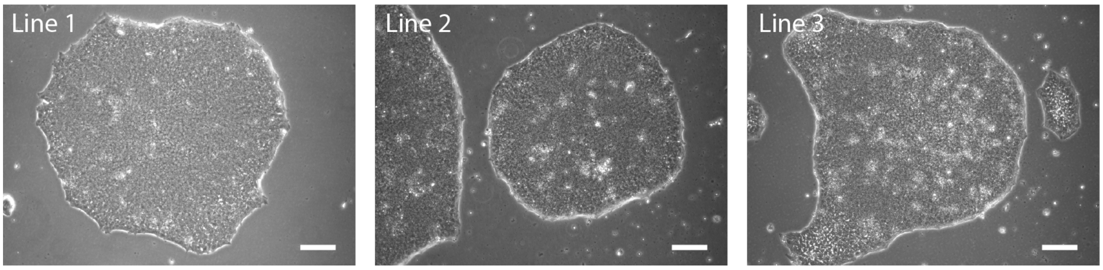

Supplementary data 1. Morphology of cGMP-compliant hPSCs maintained as colonies on Vitronectin-XF cell culture matrix. Brightfield microscopy images. Scale bars, 100µm.

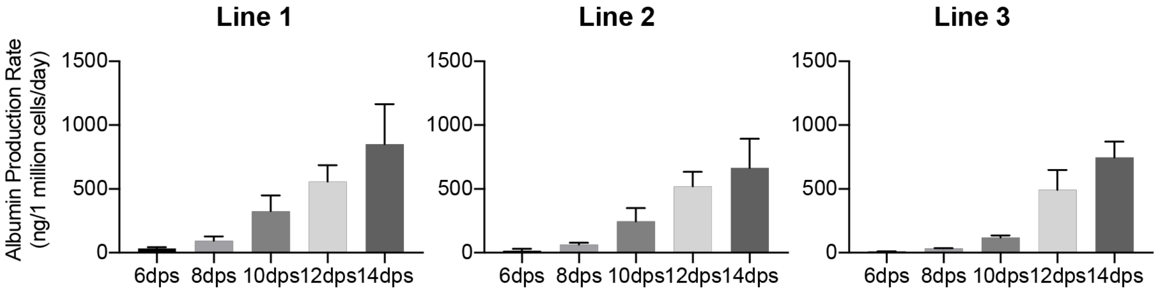

Supplementary data 2. Albumin production rate of hPSC-Heps differentiated from three lines of cGMP-compliant hPSCs. Data are mean ±SD, n = 6, 1 batch of differentiation.

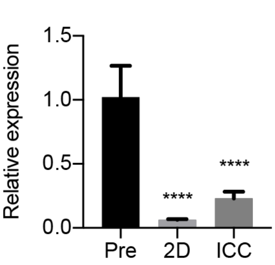

Supplementary data 3. AFP differential gene expression of hPSC-Heps. RT-PCR showing the relative gene expression of hPSC-Heps matured for 14 days within 2D and ICC

models compared to their pre-seeding population. Data shown for cell line 1. Data are mean ±SD, n=4, ****p<0.0001.

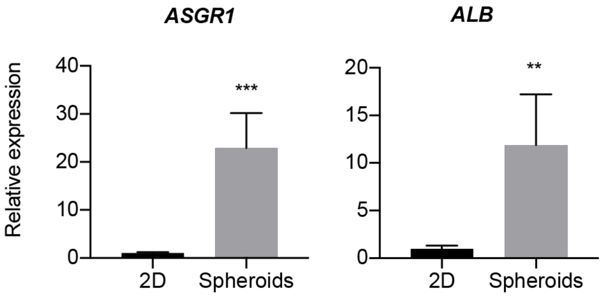

Supplementary data 4. Differential gene expression of hPSC-Heps cultured in 2D or as 3D spheroids. Gene expression, measured by RT-PCR, relative to housekeeper and normalized against the 2D condition. Data shown for cell line 3. Data are mean ±SD, n=4, **p<0.01, ***p<0.001.

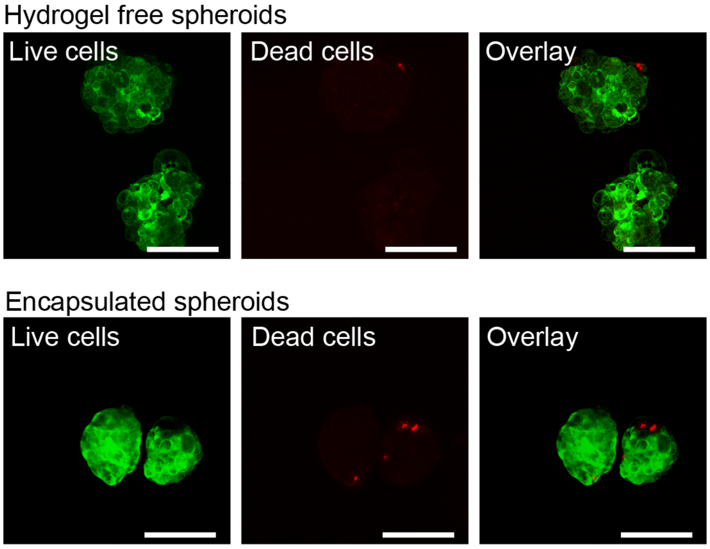

Supplementary data 5. Live/dead immunofluorescent staining of alginate encapsulated hepatocyte spheroids. Confocal images revealing the live/dead staining of hPSC-Heps cultured as hydrogel-free spheroids or within alginate microspheres 8-days post encapsulation. Data shown for cell line 2. Scale bars, 100µm.

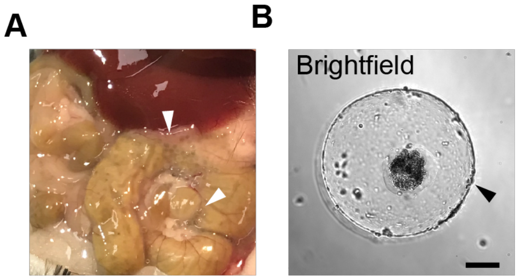

Supplementary data 6. Intraperitoneal xenotransplantation of alginate encapsulated hepatocyte spheroids into immune competent mice. (**A**) Photo of murine abdomen showing microspheres distributed throughout the peritoneal cavity with no sign of bruising, inflammation or fibrosis on 3 dpt. (**B**) Brightfield image showing an intact transplanted hepatocyte spheroid and host cell overgrowth on the surface of an alginate microsphere. Black arrowheads indicate the host cell overgrowth. Presented data collected from immune competent C57BL/6 mice. Scale bar, 100µm.

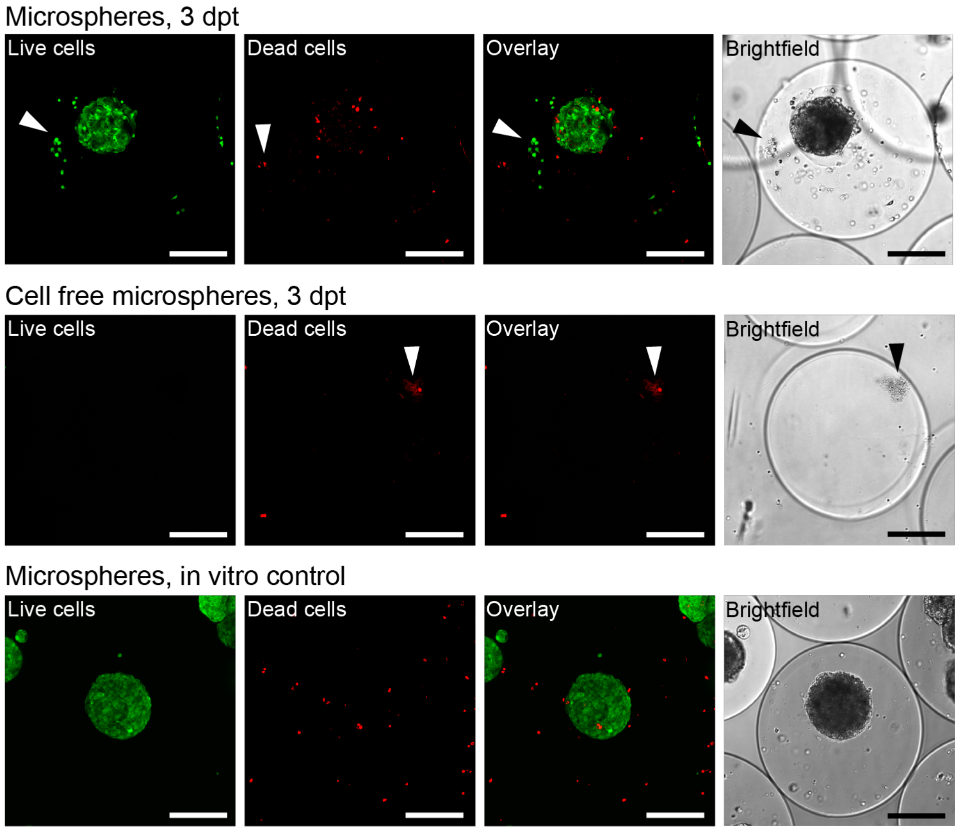

Supplementary data 7. Live/dead confocal micrographs and brightfield images of microspheres recovered on day 3 post-transplantation. Images reveal minimal transplanted cell death and host cellular adhesion on microspheres on 3dpt. Arrowheads indicate host cellular overgrowth. Data shown from cell line 2. Presented data collected from immune competent CD-1 mice. Scale bars, 100µm.

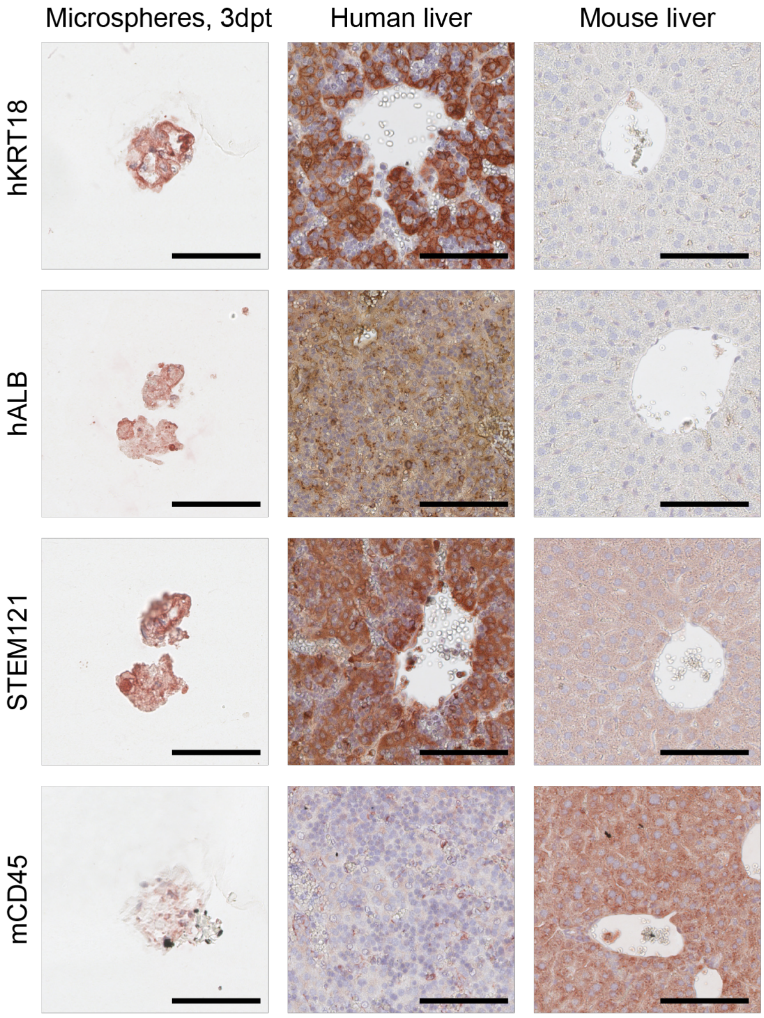

Supplementary data 8. Immunohistochemical staining of microspheres recovered from an immune competent mouse on day 3 post-transplantation. Alginate microspheres preserved the human hepatic markers (hKRT18 and hALB) of transplanted cells, and the mechanical barrier that separates human (STEM121) and host cells (mCD45). Data shown

from cell line 2. Presented data collected from immune competent CD-1 mice. Scale bars, 100μm.
